# Moving Beyond Oxford Nanopore Standard Procedures: New Insights from Water and Multiple Fish Microbiomes

**DOI:** 10.3390/ijms252312603

**Published:** 2024-11-23

**Authors:** Ricardo Domingo-Bretón, Federico Moroni, Socorro Toxqui-Rodríguez, Álvaro Belenguer, M. Carla Piazzon, Jaume Pérez-Sánchez, Fernando Naya-Català

**Affiliations:** 1 Nutrigenomics and Fish Growth Endocrinology Group, Instituto de Acuicultura Torre de la Sal (IATS, CSIC), 12595 Ribera de Cabanes, Castellón, Spain; ricardo.domingo@csic.es (R.D.-B.); federico.moroni@csic.es (F.M.); socorro.toxqui@csic.es (S.T.-R.); a.belenguer@csic.es (Á.B.); 2 Fish Pathology Group, Instituto de Acuicultura Torre de la Sal (IATS, CSIC), 12595 Ribera de Cabanes, Castellón, Spain; carla.piazzon@csic.es

**Keywords:** aquaculture, microbiota, 16S rRNA, third-generation sequencing, Oxford nanopore, MinION, native barcoding kit

## Abstract

Oxford Nanopore Technology (ONT) allows for the rapid profiling of aquaculture microbiomes. However, not all the experimental and downstream methodological possibilities have been benchmarked. Here, we aimed to offer novel insights into the use of different library preparation methods (standard-RAP and native barcoding-LIG), primers (V3–V4, V1–V3, and V1–V9), and basecalling models (fast-FAST, high-HAC, and super-accuracy-SUP) implemented in ONT to elucidate the microbiota associated with the aquatic environment and farmed fish, including faeces, skin, and intestinal mucus. Microbial DNA from water and faeces samples could be amplified regardless of the library–primer strategy, but only with LIG and V1–V3/V1–V9 primers in the case of skin and intestine mucus. Low taxonomic assignment levels were favoured by the use of full-length V1–V9 primers, though in silico hybridisation revealed a lower number of potential matching sequences in the SILVA database, especially evident with the increase in Actinobacteriota in real datasets. SUP execution allowed for a higher median Phred quality (24) than FAST (11) and HAC (17), but its execution time (6–8 h) was higher in comparison to the other models (0.6–7 h). Altogether, we optimised the use of ONT for water- and fish-related microbial analyses, validating, for the first time, the use of the LIG strategy. We consider that LIG–V1–V9-HAC is the optimal time/cost-effective option to amplify the microbial DNA from environmental samples. However, the use of V1–V3 could help to maximise the dataset microbiome diversity, representing an alternative when long amplicon sequences become compromised by microbial DNA quality and/or high host DNA loads interfere with the PCR amplification/sequencing procedures, especially in the case of gut mucus.

## 1. Introduction

Microorganisms form complex communities that play key roles in the preservation of the health and welfare of aquaculture species [1,2]. They are found in mucosal tissues such as the gut, skin, and gills, as well as in the faeces and the water surrounding the farmed fish. Real-time and precise understanding of the dynamics of these populations is key for unveiling the host–microbe network of interactions within the holobiont that directly impact aquaculture productivity and sustainability [3,4,5,6,7]. In this scenario, advances in DNA sequencing technologies, particularly Oxford Nanopore Technologies (ONTs), have emerged as a door-opener for rapid microbiome analysis through full-length 16S rRNA gene amplification [8]. Some efforts within the aquaculture field have already been conducted for designing standardised protocols for this platform. These involved the utilisation of the 16S amplification protocol recommended by ONT for the MinION^TM^ device, equipped with R9.4.1 flow cells [9,10,11,12]. With this chemistry, the amount of input DNA material and the PCR conditions (annealing temperature and number of cycles) of the ONT recommended protocol were optimised, maximising the yield of this platform using gilthead sea bream gut mucosal samples and making the technology suitable for further studies [13,14]. However, as an in-development technology, ONT is continuously experiencing equipment updates and improvements in terms of read quality and data output, which need to be taken into account while the work with thus technology advances [15,16,17]. Among these innovations, a keystone was the appearance of R10.4.1 flow cells, accompanied by an evolved chemistry (V14) and updated basecalling algorithms that improve the conversion process transforming raw electrical current data into the final nucleotide sequence [18]. This combination increased ONT read accuracy up to Q20+ quality indexes by reducing sequence anomaly detections. However, to fully exploit these novelties, users must have access to a computer equipped with a sufficiently powerful dedicated graphical processing unit (GPU) or even high-performance computing or cloud environments to manage the data produced by the highest-output ONT sequencers [19,20,21,22,23,24]. In any case, ONT innovations directly promote not only microbial analyses, but also other novel ONT applications, such as structural variation detection and methylation marks calling, enhancing a more specific taxonomic assignment and the non-redundant genome mapping of long-reads [25]. Therefore, the traditional assumption that ONT is a low-quality platform is being reconsidered, though this quality jump and the extension of the ONT catalogue of resources have not been properly tested using aquaculture-related samples yet.

Besides flow cells, the flexibility of ONT continues with alternative library preparation strategies that admit different primer choices [16,26]. The following two library preparation strategies are possible with ONT for metabarcoding studies: amplification using the 16S Barcoding Kit 24 V14 (RAP) or the ligation-based Native Barcoding Kit V14 after custom-amplicon generation (LIG). Nowadays, RAP is the standard method used for 16S microbiome studies within the aquaculture field [9,12,14]. This kit offers several clear advantages, such as a shorter protocol duration, reduced laboratory resources, and fewer pipetting or sample-handling steps. These features make it an ideal choice for in situ microbiome analysis or for use in minimally equipped laboratories. However, in well-equipped molecular biology laboratories, the LIG method offers distinct advantages that should not be overlooked [27]. LIG supports a much higher level of multiplexing, with up to 96 barcodes compared to the 24 barcodes offered by RAP. This makes the sequencing of a larger number of samples more cost-effective and time-efficient. Another major benefit of such an approach is the flexibility that it provides by separating the amplification of the targeted region from the barcoding and library preparation. This allows users to customise the targeted region length to suit their specific research needs and experiment with different primer pair modifications to achieve the best representation of the studied population [28].

The choice of primers is another well-described critical step in 16S rRNA amplification, as it directly influences the accuracy, efficiency, and reliability of microbial identification and diversity studies [29,30,31]. Certainly, unlike short-read sequencing, longer amplicons ensure low-level taxonomic assignments, usually down to the species rank [32]. However, these amplicons can be difficult to obtain when analysing DNA/RNA samples of animal and human origin, such as skin and gut mucus, among others [33]. The first reason for this is due to the host DNA, which is usually present and very abundant in these types of samples. In fact, host DNA depletion is a technique commonly used in human samples for microbiome enrichment [34], which can be also required in fish studies, as metatranscriptomic datasets of gilthead sea bream highlighted a high host/microbial RNA ratio (9:1) [35]. Secondly, DNA degradation substantially limits the fragment length that a PCR can successfully amplify. This process could be linked to sampling procedures and conditions, or even biological processes, such as the different mucosal epithelial turnover rhythms among seasons or feeding regimes [36]. When long-amplicon sequencing is not possible, the LIG procedure allows for the use of short universal primers covering certain hypervariable regions (i.e., V3–V4 or V1–V3) or ad hoc designed primers [34,37]. Traditionally, ONT has not been considered as a short-read sequencing platform, but several authors have claimed the high cost-effectiveness of attempting this approach for DNA metabarcoding studies [28,38,39]. However, due to the current lack of research using the LIG method, we are unable to know what the level of comparability is in fish-related 16S amplicon datasets using distinct primer combinations, or if short-amplicon primers also maintain the level of quality that the new R10.4.1 flow cells and basecalling algorithms offer.

Taking into account all the above findings, the higher-quality ONT equipment and LIG protocols need to be benchmarked and compared to standard procedures to choose the optimal strategy that fits the user sequencing needs. Differences in taxonomic assignment, taxa identification bias, execution time, and resource consumption remain elusive when using aquaculture-related samples. Accordingly, in order to understand whether these gaps can be filled, we aimed to optimise the 16S rRNA amplification procedures implemented in ONT when sequencing fish-associated samples of gut and skin mucosa, faeces, and water from several trials, evaluating, at the same time, the feasibility and output differences of different library preparation strategies, primers, and basecalling models.

## 2. Results

### 2.1. Assessment Strategy of ONT Performance and Sequencing Results

A total of 36 samples from a mock community (1), *E. coli* pure cultures (1), gut (4) and skin mucus (4), faeces (12), and water (15) were used to test the performance of ONT MinION^TM^ with R10.4.1 flow cells and V14 chemistry (Table 1). A mock community (ZymoBIOMICS™ Microbial Community Standard II) and a pure isolate of *E. coli* DH5α (CTRL+) were used as bacterial standards and positive controls. Gut and skin mucus samples were taken from European sea bass (*Dicentrarchus labrax*) specimens. The following two sets of water samples were taken: one from the surrounding milieu of the same European sea bass specimens and one from tanks with gilthead sea bream (*Sparus aurata*) in both the summer and winter seasons. Faeces samples were taken from gilthead sea bream (more details on sampling can be found in the Methods section). The mock, gut, skin, and water samples were processed with two different library preparation strategies (RAP and LIG), while faeces were only processed with the LIG strategy. The use of RAP only allowed for the use of full-length V1–V9 primers (27F-1492R), whereas the LIG libraries could be amplified with V1–V9 (same primer sequences as RAP), V3–V4 (341F-805R), and V1–V3 (27F-533R) primers. Available low- (FAST), high- (HAC), and super-accuracy (SUP) basecalling algorithms were applied to all the sequenced samples. After all these combinations, a total of 207 datasets were successfully obtained, with an average of 174,450 high-quality raw reads per sample, with >93% of these reads assigned to the genus level (Supplementary Table S1).

### 2.2. Amplicon Qualities

**Figure 1:**
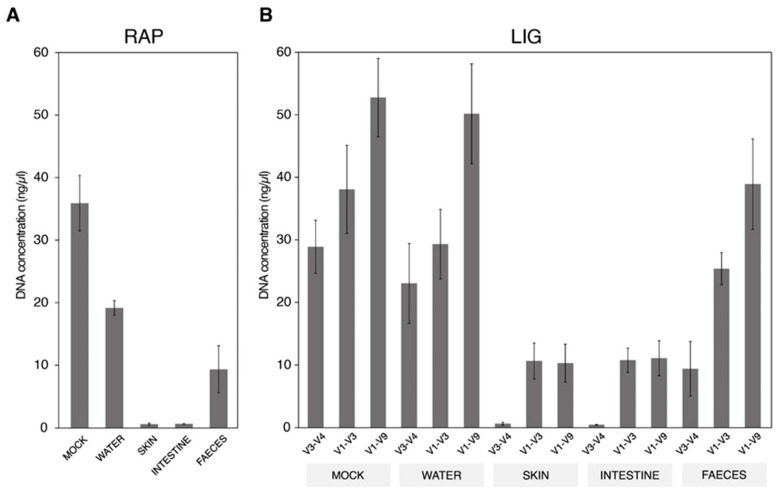
Bar plots representing the DNA concentration of PCR products in each one of the microbiomes included in this study for (**A**) RAP and (**B**) LIG methodology with different primer pairs.

Before sequencing, the PCR products obtained with the RAP and LIG methods were checked and their concentrations were measured (Figure 1). The values in the RAP method ranged from 35.9 ng/µL in the mock community and decreased in water and faeces down to 19.2 ng/µL and 9.36 ng/µL, respectively (Figure 1A). Regardless, this difference in yield was not particularly concerning, as the PCR product was sufficient for the downstream sequencing of these microbiomes. Contrarily, in the skin and intestinal mucus samples, the yields from the RAP method were notably low (less than 1 ng/µL), and the expected ~1.5 kb band was not observable in agarose gel for most of the samples, hindering the posterior sequencing. Within the LIG strategy, the yield ranged from 52.8 ng/µL in the mock community to 50.1 ng/µL in water and 38.9 ng/µL in the faeces samples when V1–V9 primers were used (Figure 1B). Interestingly, the LIG method produced amplicons with average concentrations of 10.3 ng/µL and 11.7 ng/µL in skin and intestinal mucus, with this strategy being the only one that allowed for obtaining a full-length amplicon when working with these host-associated mucus samples.

Microbiome-specific differences were observed when comparing the PCR product yields from the three primer pairs tested under the LIG method (Figure 1B). In the water and mock communities, all primer sets showed a good performance, producing amplicons with concentrations ranging from 23.0 to 50.1 ng/µL. The PCR product concentration in the faeces samples followed a similar pattern, despite their yields being lower compared with the mock and water samples (9.4–38.9 ng/µL). In contrast, the V1–V3 primers performed similarly to V1–V9 in both the skin (10.6 compared to 10.3 ng/µL) and intestine (10.8 compared to 11.1 ng/µL) samples in terms of amplicon concentration. In fact, the V1–V3 region showed a higher estimated number of copies than the V1–V9 region (30.9 compared with 10.4 fmol in skin and 31.3 compared with 11.2 fmol in intestine mucus samples). Additionally, some replicates that failed to amplify with the V1–V9 region were successfully amplified when targeting the V1–V3 region. Despite the good performance of V1–V3 and V1–V9, the amplification of the V3–V4 region was not achieved in the skin and intestine mucus samples, producing very low PCR yields and lacking the expected ~450 nt band in agarose gels in most cases, making posterior sequencing unfeasible.

### 2.3. Effects of Primers on Taxonomy Assignment

**Figure 2:**
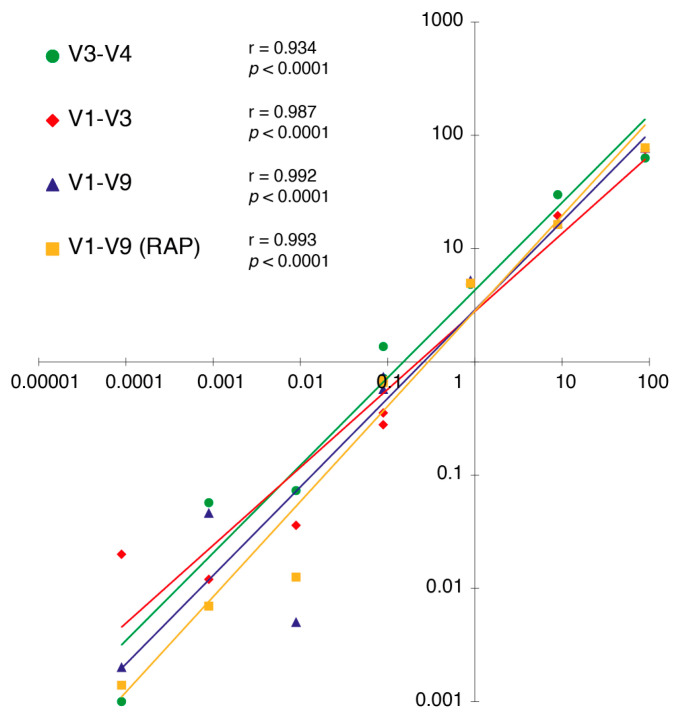
Correlation plots between the expected standard distribution of the mock community (*X* axis) and the relative abundances detected in our sequencing output (*Y* axis). A logarithmic scale was used to represent the data and a Pearson correlation coefficient (r) was calculated for each one of the library–primer combinations used in the study.

**Figure 3:**
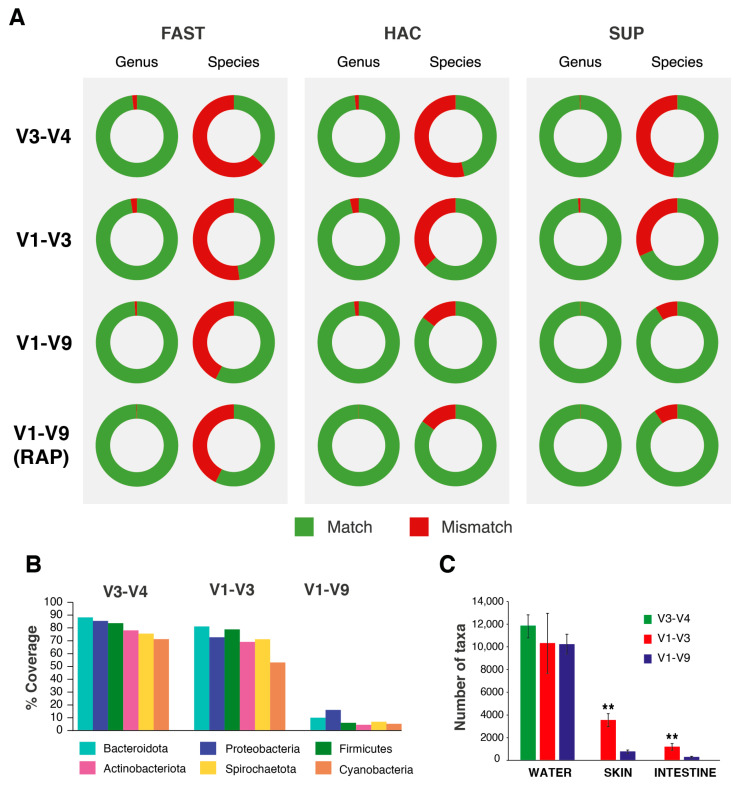
In silico and in vivo primer performance. (**A**) Pie plots representing the percentage of reads in known taxonomy samples (mock community and positive controls) assigned correctly to genus and species level in each one of the library–primer–basecalling combinations used in the study. (**B**) Bar plots showing the percentage of taxonomies of the 6 dominant phyla in aquaculture-related samples matched in SILVA after in silico hybridisation of each set of primers used in this study. (**C**) Effect of primer choice on the number of taxa found in water, skin, and intestine samples of this study (One-way ANOVA + Tukey’s post-test, ** p < 0.001).

To test the performance of the RAP and LIG methods in assigning taxa, the correlation between the theoretical composition of the mock community and the values obtained after its sequencing was tested. Regardless of the method and primer choice, a high accuracy (*r* = 0.93–0.99; *p* < 0.0001) and minimal dispersion between combinations were found (Figure 2). However, the primer choice had a strong influence on the final dataset assignment in terms of the taxonomic level resolution and taxa group representation (Figure 3). In our study, the accuracy of genus-level read assignments to the theoretical taxa in the mock communities and *E. coli* pure cultures was very high (97.5–99.9%) across all primers, library preparation strategies, and basecalling models, with few unmatched or incorrectly classified reads, even for shorter regions and less accurate basecalling models (Figure 3A). However, at the species level, the assignment accuracy dropped notably, particularly for the shorter V1–V3 (47.8–68.28%) and V3–V4 regions (37.5–51.7%). Only the full-length 16S region yielded a high percentage (57.3–91.2%) of correctly classified reads at the species level. This increase was supported by the use of more accurate models (FAST < HAC < SUP). By using V1–V9, similar matching percentages were obtained at the species level using the HAC and SUP models (85.4% and 91.2%, respectively) compared to the less accurate FAST model (57.3%), with the improvement from HAC to SUP being less pronounced than the substantial gain from FAST to HAC (Figure 3A).

To measure the in silico impact of primer choice on the database and, thus, on taxonomic assignment, we calculated the hybridisation coverages of the six most abundant phyla in the fish and water samples (Proteobacteria, Actinobacteriota, Firmicutes, Bacteroidota, Spirochaetota, and Cyanobacteria). The percentages ranged between 70 and 90% using the V3–V4 primers, and 50 and 84% using V1–V3, with the lowest value being found using the V1–V9 combination (5–20%) (Figure 3B). With this information, we investigated if the taxa number was also reduced in the real datasets, with differences depending on the microbiome. The water microbiome was not highly influenced by the primer choice, with the total number of taxonomies assigned decreasing from ~12,000 using the V3–V4 primers to ~10,000 using the V1–V3 and V1–V9 primers (*p >* 0.05). Otherwise, the skin and gut microbiomes were strongly influenced by the primer choice (*p* < 0.05), with the taxonomies retrieved decreasing from ~4000 and ~2000 with V1–V3 down to ~1000 and ~800 using the V1–V9 combination (Figure 3C).

### 2.4. Basecalling Effects on Sequencing Performance

**Figure 4:**
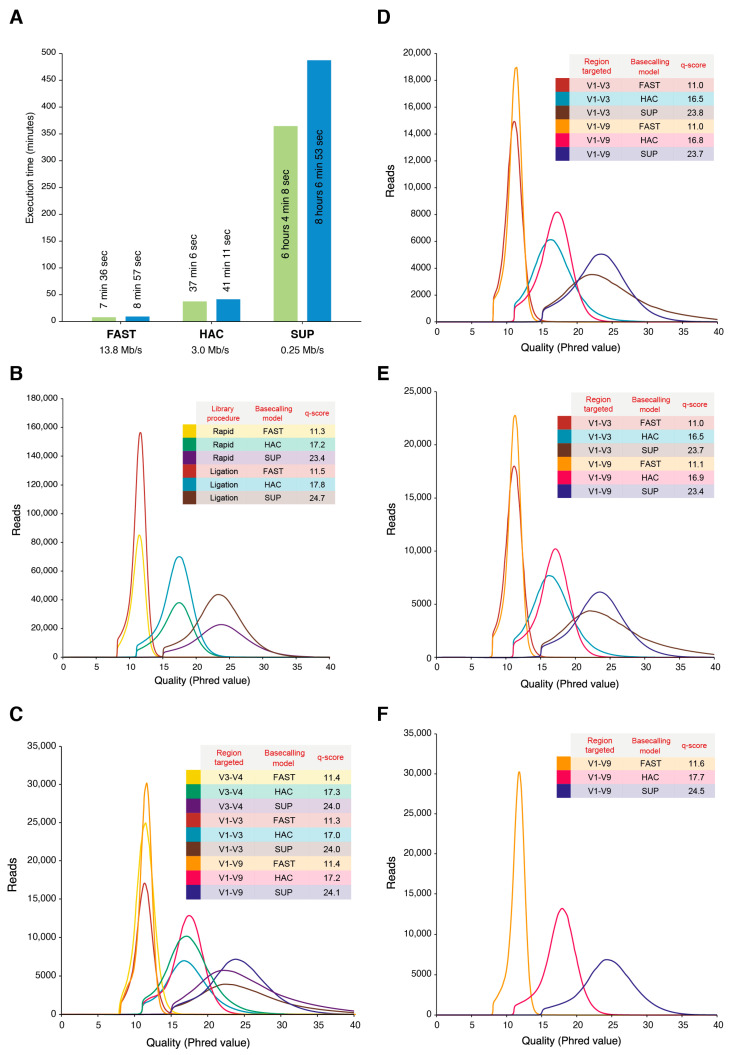
Basecalling time and quality performance. (**A**) Time of execution for each basecalling model (FAST, HAC, and SUP) used in the study for two datasets with 5.1 (green) and 7.4 (blue) Gb. Distribution of R10.4.1 reads average quality Phred score from each library preparation kit and basecalled dataset within (**B**,**C**) water, (**D**) skin mucus, (**E**) intestine mucus, and (**F**) faeces samples.

For the water, skin, intestine, and faeces microbiomes, we ran the three available basecalling algorithms implemented in the Dorado v0.7 tool with a GPU-based (NVIDIA RTX 4090) system, which showed significant differences in the execution speed. For two datasets of 5.1 and 7.4 Gb, the FAST model ran in 7–9 min, with this time increasing to 37–41 min with HAC and up to 8 h with the SUP model (Figure 4A). After basecalling and read filtering by the length and quality thresholds, the mean quality scores obtained for all the samples sequenced in this study were 11.3 for the FAST model (92.5% accuracy), 15.9 for HAC (97.5% accuracy), and 23.4 for SUP (99.5% accuracy). The q-scores were consistent across all compartments analysed. Similarly, neither the primer choice nor the library preparation methods significantly affected the sequencing accuracy, as all basecalled samples under the same model maintained similar q-score values (Figure 4B–F). The use of different basecalling models also affected the number of singletons obtained in the datasets. Singletons, considered as features with a unique count across all samples, are usually removed and as considered sequencing noise. With an increase in the sequencing accuracy, the number of global singletons was reduced, with this observed tendency being independent of the microbiome sample studied, the library preparation method, or the region targeted (Figure S1). The FAST model produced percentages of singletons ranging from 0.6% to 7.9%, showing significantly (*p* < 0.05) higher values compared with the HAC model, which ranged from 0.3% to 5.3%. The decrease in the number of singletons with the SUP model (0.2% to 4.8%) compared with the HAC model was not significant (*p* > 0.05), with HAC and SUP being very close in terms of singletons despite the big gap in sequencing quality between them.

### 2.5. Effects of Library Preparation Strategy, Primers, and Basecalling Models in Taxonomy Correlations

**Figure 5:**
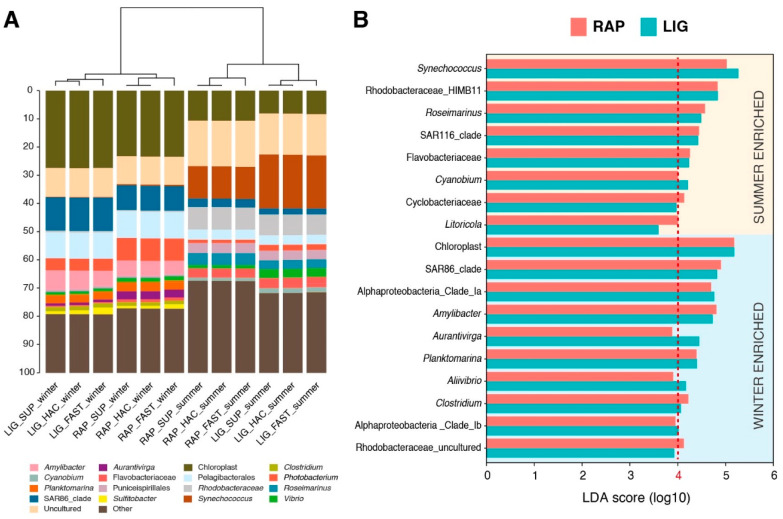
(**A**) Clustered stacked barplots showing relative abundances (sequence-based) of the most abundant genera (>1%) in summer and winter-extracted water samples. Clustering was based on Pearson correlation analysis (*p* < 0.05). Taxa with less than 1% abundance were included together in the group labelled *Other*. (**B**) Linear discriminant analysis effect size analysis performed at the level of genus representing the significant biomarkers for each group and their LDA score (log_10_).

Improvements in read quality due to basecalling algorithm updates were corroborated by analysing the taxonomic assignment in each microbiome. Using data from water samples, we were able to compare the assignment effects of the two library preparation strategies (RAP and LIG), the three primer combinations (V3–V4, V1–V3, and V1–V9), and the three basecalling models (FAST, HAC, and SUP). Regardless of the analysed variable, the unique shared taxonomies between the methods showed a very significant abundance rate in the datasets (94–99.9%) in comparison to those that were found in a particular strategy, primer, or model (Figure S2). In the first water dataset, 12 samples sequenced using each method were formed by the following two groups: one collected during the summer and the other during the winter. This selection of two distinct groups aimed to determine whether the library preparation method would yield consistent results in a real experimental setting (Figure 5). Regardless of the library preparation method, the samples from the two seasonal groups were differentiated, as shown by clustering based on Pearson’s correlation coefficient (Figure 5A). To further investigate the differences between the LIG and RAP datasets, LEfSe analysis was performed to identify microbial markers between the two seasonal groups based on data from both library methods run with the SUP basecalling model. The list of biomarkers from the LIG and RAP datasets exhibited a high degree of overlap. Each analysis identified 15 bacterial biomarkers (LDA score threshold = 4, α < 0.05) at the genus level (Figure 5B), with 12 of these markers being above the significance threshold in both protocols and only 3 showing discrepancies. However, upon closer examination of the LDA scores for the divergent markers, these scores were found to be just above the threshold in one dataset and slightly below in the other, demonstrating a strong consistency in the results from both datasets (Figure 5B).

**Figure 6:**
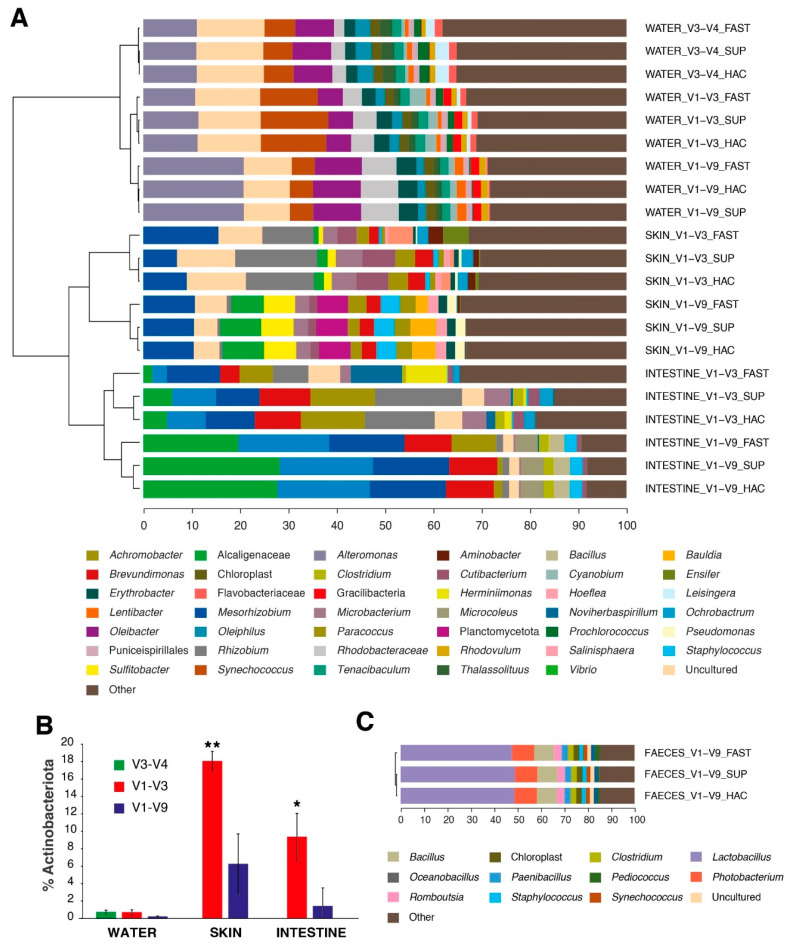
(**A**) Clustered stacked barplots showing relative abundances (sequence-based) of the most abundant genera (>1%) of the water, skin, and mucus. Clustering was based on Pearson correlation analysis (*p* < 0.05). Taxa with less than 1% abundance were grouped together in the group labelled *Other*. (**B**) Effect of primer choice on the number of Actinobacteriota taxa found in water, skin, and intestine samples of this study (Kruskal–Wallis + Dunn’s post-test, * *p* < 0.05; ** *p <* 0.001). (**C**) Clustered stacked barplots showing relative abundances (sequence-based) of the most abundant genera (>1%) of the faeces.

The remaining samples of water, skin, and intestinal mucus included in this study were used in the experimental trial using European sea bass. These samples were used to study the differences in the taxonomic assignment in different microbiomes due to the primer choice or the basecalling model used. All successfully amplified and sequenced samples, including their three basecalling alternatives, were put together and clustered based on their similarity represented by Pearson’s correlation coefficient. The compartment variable was, as expected, the first clustering observed, with water microbiome being separated from the skin and intestine mucus microbiomes (Figure 6A). In the water samples, where all the primers were tested, V3–V4 showed a very high correlation with the V1–V9 region (*r* = 0.86). However, a higher correlation was obtained when comparing the V1–V3 region with the full-length 16S (*r* = 0.91). In host mucosal samples, the comparison of the V1–V3 and V1–V9 regions showed a correlation coefficient of ~0.5. Despite these low values, among the most abundant taxa (>1%) represented in Figure 6A, 11 out of 16 taxa in gut mucus and 10 of 15 taxa in skin mucus varied less than 3% in the final dataset. The greatest changes were observed in the Actinobacteriota representations. Indeed, the water microbiome barely contained < 1% of this taxonomic group. Otherwise, the skin and gut mucus microbiomes accounted for 18% and 10% of the Actinobacteriota-assigned reads with the V1–V3 primers, whereas this number decreased significantly (*p* < 0.05) to 6% and 2%, respectively, with the use of the V1–V9 primers (Figure 6B). Reads assigned to *Cutibacterium* and *Microbacterium* were the most differentiated Actinobacteriota taxa between the primer combinations. In the lower level of clustering, fewer differences were observed between basecalling models compared to primer pairs. Regardless of the microbiome or the amplified region, and including faeces samples (Figure 6C), a consistent pattern was noted. The samples basecalled with the HAC and SUP models showed a very strong correlation (r > 0.93 in all samples). In contrast, the samples basecalled with the FAST model were less correlated with the more accurate models (r > 0.81 in all samples). As a result, the SUP and HAC basecalled samples clustered together and were distinct from the FAST basecalled samples across all microbiomes and regions sequenced.

## 3. Discussion

The third-generation ONT sequencing platform allows for the rapid and in-house characterisation of microbiomes as a key method to advance the understanding of hologenomic relationships between hosts and microorganisms [3,4,6,7,40]. In this study, we report the outcomes after using the ONT MinION^TM^ device equipped with R10.4.1 flow cells for metataxonomic data through the amplification of the 16S rRNA gene. To pursue this issue in a comprehensive manner, we first scanned the catalogue of options that ONT offers in order to benchmark the different library preparation strategies, depicting differences depending on the primer and basecalling model choice in water and fish-related gut, skin, and faeces samples (Table 1). In this sense, it must be noted that the RAP method is, nowadays, the reference protocol for metabarcoding studies in aquaculture-related samples [12,14]. However, the utilisation of the LIG method has been tested successfully in environmental DNA (eDNA) metabarcoding studies using short reads as an accurate and cost-effective methodology [28,41]. Recent studies have also successfully used full-length 16S amplification procedures under the LIG methodology with human samples and bacterial cultures [42,43]. In contrast, low yields using the RAP strategy have already been noted in different fish species, including gilthead sea bream and rainbow trout [12,14]. In fact, to obtain enough amplicon amounts for microbiome sequencing, these last authors increased the input of the DNA template up to 1000 ng and modified the PCR conditions. Despite this, we obtained herein very low amplification yields (<1 ng/µL) using the RAP methodology in skin and intestinal mucus samples, greatly hindering their posterior sequencing (Figure 1A). At this point, the LIG strategy emerged as a viable alternative to the standard procedure, increasing the yields of the PCR products compared to RAP, especially in host-associated samples (Figure 1B). Furthermore, the feasibility of LIG was reinforced by taxonomy assignments that were closely correlated with those achieved with RAP when using mock (Figure 2) and real (Figure 5A) datasets, without affecting low-level taxonomy assignment (Figure 3A), data noise (Figure S2), or quality loss (Figure 4B).

Although the primer combination, enzymes, and input template were replicated exactly in RAP and LIG, the primers provided with the RAP kit also contain specific barcoding sequences and attachment chemistry, which facilitates the subsequent addition of sequencing adapters without the need for ligation reactions [44]. It can be hypothesised that, at some point, these elements can reduce the PCR yield, but the actual reasons for this are not understood yet. To try to solve this problem, some authors have performed the two following separate PCR reactions: an initial PCR with conventional primers to amplify the target region, followed by a second PCR using the barcoded primers of the 16S kit to prepare the sequencing libraries [9]. However, the use of two consecutive PCR reactions with the same primer sequence can introduce artifacts and lead to PCR bias, which needs to be considered during the data analysis step [45,46]. Furthermore, the reduced yield of the RAP method was particularly concerning in mucosal tissues, where host DNA usually represents a major fraction of the total sample compared to bacterial DNA [47,48,49,50]. Then, the results of the present study reinforce the already posed idea that RAP works better with environmental samples, high-quality samples, or samples less contaminated with host DNA [14], while LIG extends the possibilities for sequencing challenging samples and precludes their loss, as occurs when RAP amplification is not possible. Altogether, this work represents the first study to optimise the use of the ONT LIG strategy in fish- and water-related samples, as well as the first direct comparison between the LIG and RAP methods for microbiome analysis. In this sense, it is noteworthy that our findings highlight a strong correlation between the two methodological approaches (Figure 5B), making it feasible that the choice of method can depend exclusively on the resources and analytical needs of each experiment.

In addition to the above findings, the use of the LIG strategy opens the door to both long- and short-read sequencing in ONT with different primer combinations that the user can define ad hoc. In this study, the V1–V3 and V1–V9 regions could be amplified in all mock, fish, and water microbiomes, but with our experimental conditions (i.e., PCR conditions and ONT-recommended polymerase for RAP), the use of the V3–V4 primers remained limited to mock, water, and faeces samples (Figure 1B and Figure 2). At the same time, an increase in the amplicon length (V1–V9) led to the assignment of lower taxonomic levels (genus and species) in our final datasets (Figure 3A), confirming the actual advantage of sequencing with ONT, as already confirmed by several studies [51,52,53]. However, this taxonomic assignment performance was accompanied by lower in silico hybridisation using long primers, which was evidenced herein when we analysed the skin and gut mucus microbiomes (Figure 3B,C). The hybridisation of the chosen primers in the sample DNA would directly impact amplicon generation and, then, posterior mapping to the reference database. In this study, we used the SILVA v138.1 database, one of the most referenced and updated 16S rRNA databases, with almost 1.5 M non-redundant entries [54]. However, the high sequence similarity between SILVA and the rest of the main databases (i.e., RDP and Greengenes) suggests a more than probable parallelism [55]. Thus, good hybridisation should be promoted in the first instance to avoid taxa under-representations [56,57], which was the case for our gut and skin mucus datasets, where V1–V9 clearly yielded a decrease in the abundances of the Actinobacteriota phylum (Figure 6B). In fact, this trend has already been observed by several authors, who reported an Actinobacteriota taxa under-estimation and nucleotide mismatch in primer hybridisation [50,58]. Taxa belonging to the Actinobacteriota phyla have been isolated from fish gut and related to gut and skin functionality in fish and aquaculture species, as seen in diverse studies regarding fish genetics, ecology, and nutrition [4,59,60,61]. Thus, solving this limitation is key for an accurate and complete representation of aquaculture microbiomes and holobionts, and will allow for developing ONT’s full potential. Some steps are being taken towards this goal, such as the design of ad hoc primers and databases [50,62] or the testing of the ~4.3 kb 16S-ITS-23S region from the *rrn* operon as a better microbial marker [63]. As long-read sequencing continues to evolve, targeting alternative regions of the 16S rRNA gene could serve as a viable option to ensure the accurate characterisation of diverse microbiomes. Indeed, the use of V1–V3 has previously depicted a resolution comparable to that of full-length 16S rRNA sequences analysed over diverse human microbiomes with the long-read PacBio platform [52,64,65]. In this line, our results suggest that the V1–V3 region can serve as a reliable alternative to the V1–V9 region, offering a higher representation of the phylum Actinobacteriota (Figure 6B) and a greater consistency in amplifying all types of microbial samples. Additionally, the sequencing of a shorter region could also lead to a very cost-effective sequencing procedure on ONT platforms [28] for monitoring microbial communities when the species level is not required, as the taxonomic resolution remains the major trade-off compared with the V1–V9 region. Certainly, arriving to a species-level resolution is not widespread in 16S analysis, as most users often collapse their results to the genus level. In this sense, the use of shorter primers can help to raise the microbial diversity in final datasets. Therefore, based on our results, we consider the amplification of the V1–V3 region, using a conventional PCR with subsequent attaching barcodes and sequencing adapters through the LIG strategy, as an alternative to long-read amplicons.

Lastly, it must be noted that the ONT procedure is based on the foundation that an electric current potential is transformed into a nucleotide sequence in a basecalling process. For this reason, the major efforts of ONT have been addressed to improve basecalling algorithms and tools during the last few years [44]. This work has paid off, and the mean q-score achieved with the most advanced SUP model represented, in our work, a substantial improvement over previous chemistry iterations, surpassing the barrier of a q-score of 20 or a 99% accuracy, regardless of the type of library preparation and the primer combination (Figure 4B–F). However, this increase in accuracy comes at a significant computational cost. Running the SUP model requires considerably more hardware resources than the FAST and HAC models, as occurred with the fish samples and R9.4.1 chemistry [12]. This increased computational requirement may not be a major issue when adequate resources are available, as was the case in this study, where a GPU-based computer was sufficient to perform the real-time basecalling of the MinION^TM^ output (Figure 4A). However, for systems with low-spec GPUs, or without a dedicated one, the execution time of more accurate models may increase considerably, surpassing the sequencing run time by far or even making it directly impracticable [18,66]. In terms of taxonomy assignment, the jump in read quality due to the evolution of basecalling models did not have a strong effect, and was accompanied by a higher species-level assignment (Figure 3A). Interestingly, this increase was very comparable between the HAC and SUP models, the two basecalling models that performed equally in this study. Indeed, the singleton number obtained using HAC and SUP was significantly lower compared to that with FAST (Figure S1), reducing the noise in the dataset. Clustering also reinforced this parallelism, rendering HAC and SUP taxonomy assignments always with correlation coefficients of 0.99 (Figure 5A and Figure 6A,B). Therefore, although the SUP model is recommended to maximise the quality of produced data, the HAC and SUP models remain closely related in terms of sequencing noise and taxonomic assignment in metabarcoding studies, offering HAC an increased speed in result generation and an adequate cost-effective alternative when computational resources are not available.

**Figure 7:**
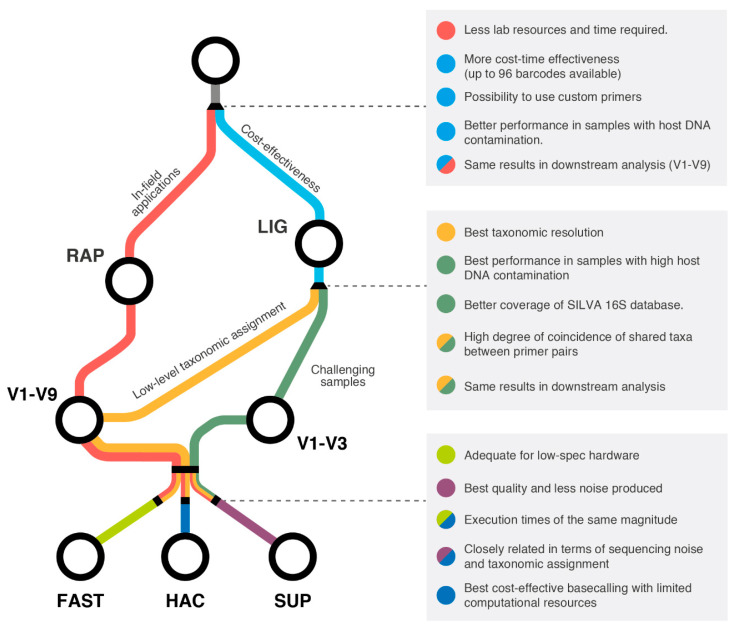
Proposed framework for decision making of the most cost-effective protocol when using ONT devices, including pros and cons of the different catalogue resources tested in this study.

In conclusion, this study provides a comprehensive benchmarking of the variables involved in 16S microbiome analysis on the ONT platform (Figure 7). The LIG and RAP library preparation methodologies performed equally, and the choice between them only depends on the specific experimental requirements. For challenging samples, targeting the V1–V3 region serves as a practical alternative to V1–V9, yielding an increased diversity at the genus level. Finally, the HAC model offers a cost-effective alternative to SUP for basecalling metabarcoding sequencing data when computational resources are limited. Altogether, these guidelines can serve as a reference for more accurate, efficient, and tailored sequencing approaches for diverse aquaculture microbiome studies.

## 4. Materials and Methods

### 4.1. Ethics Statement

The fish manipulation and tissue collection were carried out according to the Spanish (Royal Decree RD53/2013) and current EU (2010/63/EU) legislation on the handling of experimental fish. All procedures were approved by the Ethics and Animal Welfare Committee of the Institute of Aquaculture Torre de la Sal (IATS-CSIC, Castellón, Spain), CSIC Ethics Committee (permission 1135/2021), and Generalitat Valenciana (permission 2021–VSC-PEA-0192).

### 4.2. Mock Community, Water, and Fish Samples

For bacterial standard samples, the ZymoBIOMICS™ Microbial Community Standard II (Log Distribution) (Zymo Research, Tustin, CA, USA) was used in this study. This mock community is composed of eight bacteria strains and two yeasts (not applicable in 16S rRNA gene studies), following a logarithmic distribution of theorical concentrations based on genomic DNA, as follows: *Listeria monocytogenes* (89.1%), *Pseudomonas aeruginosa* (8.9%), *Bacillus subtilis* (0.89%), *Escherichia coli* (0.089%), *Salmonella enterica* (0.089%), *Lactobacillus fermentum* (0.0089%), *Enterococcus faecalis* (0.00089%), and *Staphylococcus aureus* (0.000089%). Additionally, another standard consisting of bacterial DNA from a pure isolate of *E. coli* DH5α was used as a positive control for PCR and was also sequenced as a negative control, reflecting possible contaminations during the library preparation. The DNA extraction of both standards was carried out following the same procedure, using the High Pure PCR Template Preparation Kit (Roche, Basel, Switzerland) with a previous step of enzymatic lysis with lysozyme (250 μg/mL; 15 min; 37 °C) [67].

Samples from four different microbiomes were obtained for this work, as follows: water, skin and anterior intestine mucus, and faeces. Animal-derived samples were obtained from fish grown under natural photoperiod and temperature conditions fed with standard diets at the experimental facilities of IATS (40°5′ N; 0°10′ E). Water samples were collected directly from the fish tanks with sterile glass bottles (1 L), and bacterial biomass was then filtered with a manifold system using mixed cellulose ester filters with a pore size of 0.22 μm. DNA from the water samples was extracted using the DNeasy PowerSoil Pro kit (Qiagen, Hilden, Germany) following the manufacturer’s instructions. The filters were transferred to sterile Petri dishes, cut into small pieces, and submitted to mechanical lysis with the ceramic bead tubes provided in the kit, using the FastPrep 24 homogenizer (MP Biomedicals, Irvine, CA, USA) at 6 m/s for 30 s. Skin mucus samples were obtained by gently scrubbing the surface of the fish skin with a sterile microscope slide in favour of the scales, and then transferred to sterile 1.5 mL tubes and stored at −80 °C until DNA extraction. A portion of the anterior intestine (~2 cm) was opened and washed with sterile Hank’s balanced salt solution to discard non-autochthonous bacteria, collecting only adherent bacteria by scraping off intestinal mucus with the blunt edge of a sterile scalpel. DNA was extracted from the skin and anterior intestine mucus using the High Pure PCR Template Preparation Kit (Roche, Basel, Switzerland), as described above. Faeces were obtained extracting the whole intestine and squeezing out all the intestinal content directly into a sterile tube with the aid of sterile forceps. DNA from the faeces was extracted using the DNeasy PowerSoil Pro kit (Qiagen, Hilden, Germany) using 250 mg of faeces as a starting material. The concentration and quality of the extracted DNA were determined in all the samples with Nanodrop 2000c (Thermo Fisher, Waltham, MA, USA).

### 4.3. Rapid 16S Barcoding Kit Sequencing

The complete V1–V9 region rRNA was amplified and barcoded using the 16S Barcoding kit 24 V14 (RAP; SKQ-16S114.24) following the manufacturer’s protocol (version 16S_9199_V114_revB_06Dec2023), using provided 27F-1492R barcoded primers (Table 2) and including modifications of input DNA and PCR conditions optimised elsewhere for this kit [14]. PCR products were visualised in agarose gel (1% *w*/*v* TAE buffer) to check for the presence of the specific band of ~1500 bp, and DNA concentrations were determined by fluorescence using PicoGreen dye (Thermo Fisher, Waltham, MA, USA). Libraries were pooled, purified using Agencourt AMPure XP beads (Backman Coulter, Brea, CA, USA), and loaded in an ONT MinION^TM^ (Oxford Nanopore Technologies, Oxford, UK) sequencing device following the manufacturer’s protocol, then sequenced using a flow-cell R10.4.1 (FLO-MIN114). Sequencing data were acquired using MinKNOW v24.02.6 software.

### 4.4. Ligation Sequencing of Amplicons

The Native Barcoding kit 96 V14 (LIG; SQK-NBD114.96) was used to amplify three different hypervariable regions of the 16S rRNA gene (V3–V4, V1–V3, and V1–V9), using universal primers and adapted PCR conditions (Table 2). All PCRs were performed in a total volume of 25 µL; 12.5 µL of Long Amp Hot Start Taq 2x Master Mix (New England Biolabs, Ipswich, MA, USA), 1 µL of each primer (9 µM), and 10.5 µL of template DNA at the specific concentration for each type of sample made up with Ultrapure DNase/RNase-Free Distilled Water (Invitrogen, Waltham, MA, USA). Negative controls were included to check for possible contamination. PCR products were purified using Agencourt AMPure XP beads (Backman Coulter, Brea, CA, USA) using a beads/sample ratio of 0.4 for full-length amplicons and 0.6 for short-amplicon fragments. After clean-up, the amplicons were visualised in agarose gel (1% *w*/*v* TAE buffer) to ensure the amplification of the desired length fragment (V3–V4 ~ 460 bp; V1–V3 ~ 500 bp; and V1–V9 ~ 1500 bp), and the DNA concentration was quantified using Picogreen (Thermo Fisher, Waltham, MA, USA). Libraries from each length were separately sequenced in a R. 10.4.1 flow cell after flushing it with the Flow Cell Wah Kit (EXP-WSH004), always using unique barcodes in each flow cell to avoid cross-contamination between subsequent runs. Sequencing data were acquired using the MinKNOW v24.02.6 software.

### 4.5. Basecalling and Bioinformatic Analysis

Raw sequencing POD5 files from all the runs performed in this work were basecalled using Dorado v0.7 (https://github.com/nanoporetech/dorado; last accessed: 25 August 2024) using a computer equipped with an Nvidia RTX 4090 24 GB GPU. All the samples were basecalled using all the three available models in their latest versions, as follows: FAST (dna_r10.4.1_e8.2_400bps_fast@v5.0.0); HAC (dna_r10.4.1_e8.2_400bps_hac@v5.0.0); and SUP (dna_r10.4.1_e8.2_400bps_sup@v5.0.0). The basecalled samples were then demultiplexed and trimmed from barcodes and adapters using Dorado v0.7. Resulting BAM files were converted into FASTQ format using samtools v1.10 [68]. These raw basecalled FASTQ files were uploaded to the Sequence Read Archive (SRA) under the Bioproject accession number PRJNA1177626 (BioSample accession numbers: SAMN44444729-935). Raw reads were filtered using Chopper v.0.8.0 [69], using different length thresholds depending on the region targeted (V3–V4 = 300–550 bp; V1–V3 = 300–600 bp; and V1–V9 = 1200–1800 bp). In the same line, samples were filtered for quality with different minimum thresholds based on the basecalling model used in each case (FAST, q = 8; HAC, q = 11; and SUP, q = 15). These variable quality values were calculated to reach a uniform number of reads in all the samples from one specific tissue, regardless of the basecalling model used. Quality and length metrics were obtained for each sample using NanoPlot v1.42.0 [69]. Filter reads were then taxonomically assigned with minimap2 v2.28-r1209 [70] using SILVA v138.1 as a reference database [71].

### 4.6. Data Analysis

In order to evaluate the potential performance of the primer pairs, an in silico PCR test was performed using TestPrime v1.0, which computes the coverages for each taxonomic group in all the taxonomy entries found in the SILVA v138.1 database [72]. Differences in the number of taxa were obtained from each basecalling model by one-way ANOVA (Tukey’s post-test, *p <* 0.05), while the number of singletons and phylum abundance were analysed by Kruskal–Wallis test (Dunn’s post-test, *p <* 0.05) using SigmaPlot v14 (Systat Software Inc., Chicago, IL, USA). The normality of the data was verified by the Shapiro–Wilk test. Analysis of the bacterial compositional data was performed in R v4.3.0 using the packages *phyloseq* v1.44.0 [73], *corrplot* v0.94 [74], and *microbiomeMarker* v1.9.0 [75]. Pearson’s correlations (*p* < 0.05) were used for the comparison of the bacterial compositional data between samples obtained using different primers, library preparation strategies, and basecalling models. The samples were clustered depending on their Pearson correlation coefficients. To determine the bacteria genera that most likely explainthe differences between seasons within the water samples, a linear discriminant analysis (LDA) effect size (LEfSe) was conducted with *microbiomeMarker* v1.9.0 (LDA cutoff = 4, Wilcoxon cutoff = 0.05).

## Figures and Tables

**Table 1:** Tissues and experimental conditions of the ONT data used in this study. Conditions in red failed at some experimental point, hindering the sequencing of the corresponding samples.

Tissue/ Standard	Sp.	Samples	Libr. Strategy	Primers	Basecalling
Mock	MOCK	1	RAP; LIG	V3–V4; V1–V3; V1–V9	FAST; HAC; SUP
CTRL+	*E. coli* DH5α	1			
Water	-	12	RAP; LIG	V1–V9	
	-	3	LIG	V3–V4; V1–V3; V1–V9	
Skin	ESB	4	RAP; LIG	V3–V4; V1–V3; V1–V9	
Intestine	ESB	4			
Faeces	GSB	12	LIG	V1–V9	

RAP: 16S Barcoding Kit 24 V14; LIG: Native Barcoding Kit V14 after custom-amplicon generation; VX-X: Hypervariable regions overlapped with primer set; FAST: Fast-accuracy basecalling model; HAC: High-accuracy basecalling model; SUP: Super-accuracy basecalling model; MOCK: ZymoBIOMICS™ Microbial Community Standard II; CTRL+: pure isolate of *E. coli* DH5α; ESB: European sea bass; GSB: gilthead sea bream.

**Table 2:** Sequences of the forward (F) and reverse (R) primers used for the amplification of the hypervariable regions of the 16S rRNA gene, as well as Denaturation (D), Annealing (A), and Extension (E) temperatures induced during PCR amplification with each primer pair.

16S Gene Region	Primer Pair	PCR Conditions
V1–V3 (27F-533R)	F: AGA GTT TGA TCM TGG CTC AG R: TTA CCG CGG CKG CTG GCA CG	D: 95 °C 1 min A: 30 × [95 °C 20 s, 56 °C 30 s, 65° 1 min] E: 65 °C 5 min
V3–V4 (341F-805R)	F: CCT ACG GGN GGC WGC AG R: GAC TAC HVG GGT ATC TAA TCC	D: 95 °C 1 min A: 30 × [95 °C 20 s, 56 °C 30 s, 65° 1 min] E: 65 °C 5 min
V1–V9 (27F-1492R)	F: AGA GTT TGA TCM TGG CTC AG R: CGG TTA CCT TGT TAC GAC TT	D: 95 °C 1 min A: 30 × [95 °C 20 s, 52 °C 30 s, 65° 2 min] E: 65 °C 5 min

## Data Availability

All the basecalled data (FASTQ files) used in this work were loaded in the Sequence Read Archive (SRA) under the Bioproject accession number PRJNA1177626 (BioSample accession numbers: SAMN44444729-935). Raw sequencing data prior to basecalling (POD5 files) can also be obtained upon request.

## Supplementary Materials

The following supporting information can be downloaded at: https://www.mdpi.com/article/10.3390/ijms252312603/s1.
