# Supplementary material for: Moving Beyond Oxford Nanopore Standard Procedures: New Insights from Water and Multiple Fish Microbiomes
Source: Int J Mol Sci. 2024 Nov 23;25(23):12603. doi: 10.3390/ijms252312603 (PMC11641276; doi:10.3390/ijms252312603)
Supplement: Supplementary file 1 [file ijms-25-12603-s001.zip › ijms-3315508-supplementary figures.pdf]

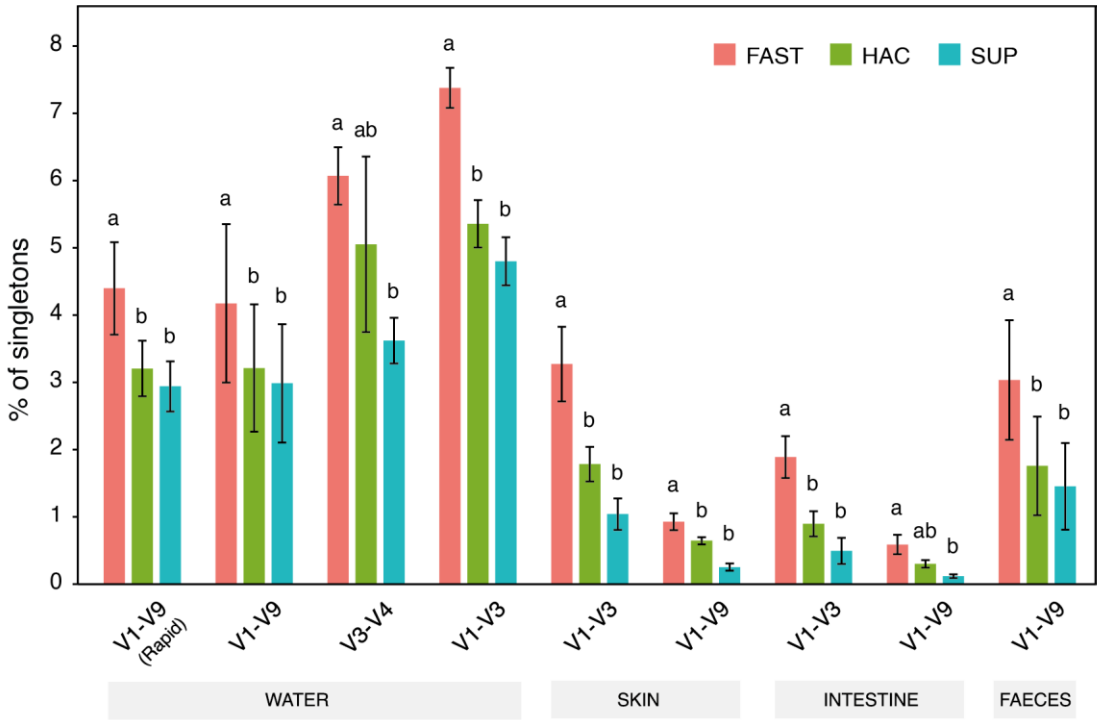

Figure S1. Bar plots representing the percentage of singletons found in the final dataset of each of the microbiomes analysed in this study, using the different library preparation strategies and primer combinations.

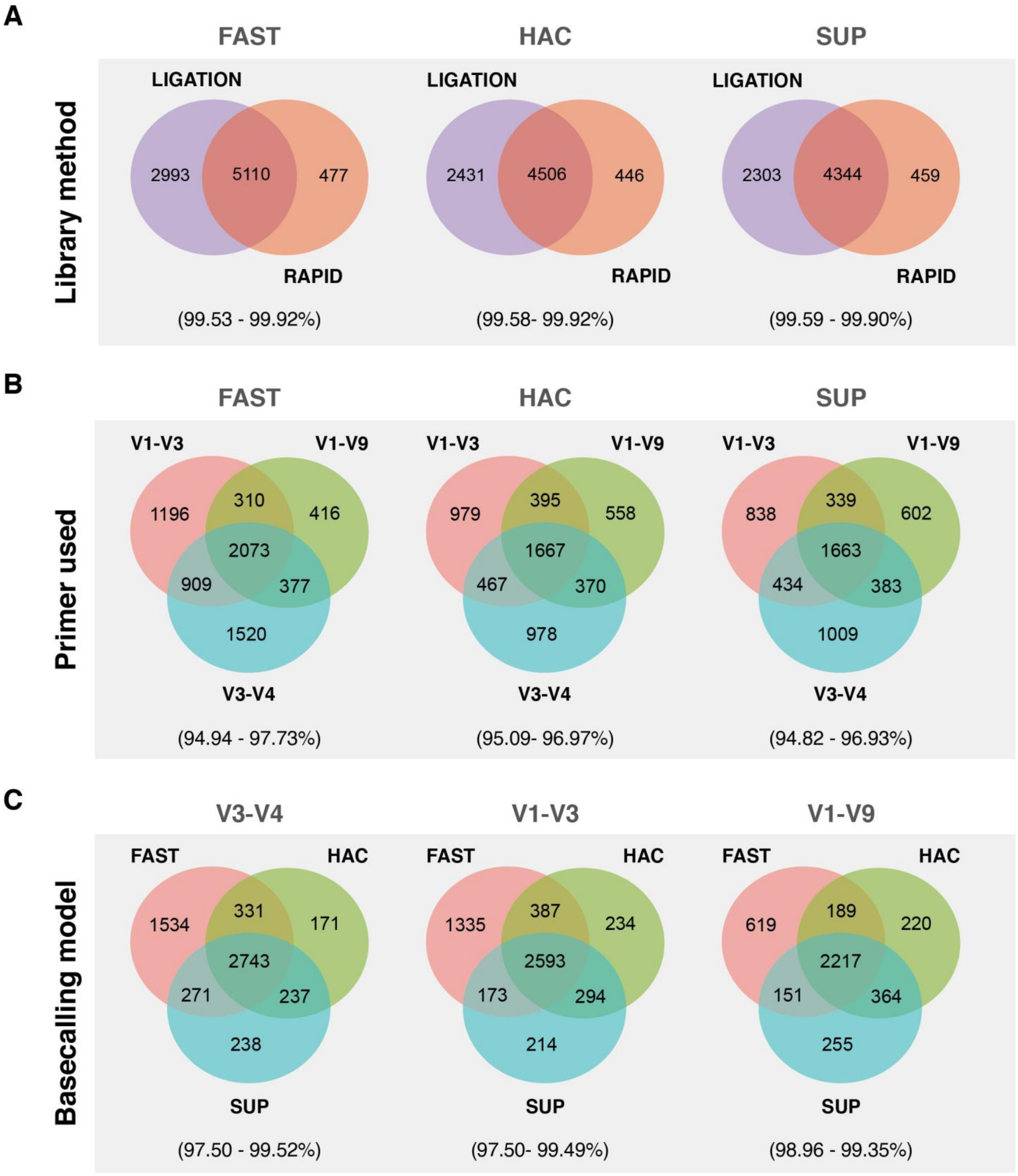

Figure S2. Pie plots representing the number of shared and differential taxa that can be found using the different basecalling models depending on **(A)** Library preparation method and **(B)** primer combination, as well as **(C)** the number of taxa found with each primer combination using the three different basecalling models.
